# Genetic structure of major depression symptoms across clinical and community cohorts

**DOI:** 10.1101/2023.07.05.23292214

**Published:** 2023-07-07

**Authors:** Mark J Adams, Jackson G Thorp, Bradley S Jermy, Alex S F Kwong, Kadri Kõiv, Andrew D Grotzinger, Michel G Nivard, Sally Marshall, Yuri Milaneschi, Bernhard T Baune, Bertram Müller-Myhsok, Brenda WJH Penninx, Dorret I Boomsma, Douglas F Levinson, Gerome Breen, Giorgio Pistis, Hans J Grabe, Henning Tiemeier, Klaus Berger, Marcella Rietschel, Patrik K Magnusson, Rudolf Uher, Steven P Hamilton, Susanne Lucae, Kelli Lehto, Qingqin S Li, Enda M Byrne, Ian B Hickie, Nicholas G Martin, Sarah E Medland, Naomi R Wray, Elliot M Tucker-Drob, Estonian Biobank Research Team, Major Depressive Disorder Working Group of the Psychiatric Genomics Consortium, Cathryn M Lewis, Andrew M McIntosh, Eske M Derks

**Affiliations:** 1 Division of Psychiatry, University of Edinburgh, Edinburgh, UK; 2 Mental Health and Neuroscience, QIMR Berghofer Medical Research Institute, Brisbane, QLD, AU; 3 Institute for Molecular Medicine Finland, University of Helsinki, Helsinki, FI; 4 MRC Integrative Epidemiology Unit, University of Bristol, Bristol, UK; 5 Estonian Genome Centre, Institute of Genomics, University of Tartu, Tartu, EE; 6 Department of Psychology and Neuroscience, University of Colorado at Boulder, Boulder, CO, US; 7 Institute for Behavioral Genetics, University of Colorado at Boulder, Boulder, CO, US; 8 Department of Biological Psychology, Vrije Universiteit Amsterdam, Amsterdam, NL; 9 Centre for Genomic & Experimental Medicine, Institute of Genetics and Cancer, University of Edinburgh, Edinburgh, UK; 10 Department of Psychiatry, Amsterdam Public Health and Amsterdam Neuroscience, Amsterdam UMC, Vrije Universiteit Amsterdam, Amsterdam, NL; 11 Department of Psychiatry, University of Melbourne, Melbourne, VIC, AU; 12 Florey Institute of Neuroscience and Mental Health, University of Melbourne, Melbourne, VIC, AU; 13 Department of Psychiatry, University of Münster, Münster, NRW, DE; 14 Department of Translational Research in Psychiatry, Max Planck Institute of Psychiatry, Munich, BY, DE; 15 Munich Cluster for Systems Neurology (SyNergy), Munich, BY, DE; 16 Institute of Population Health, University of Liverpool, Liverpool, UK; 17 Department of Biological Psychology & Amsterdam Public Health Research Institute, Vrije Universiteit Amsterdam, Amsterdam, NL; 18 Department of Psychiatry & Behavioral Sciences, Stanford University, Stanford, CA, US; 19 Social, Genetic and Developmental Psychiatry Centre, King’s College London, London, UK; 20 NIHR Maudsley Biomedical Research Centre, King’s College London, London, UK; 21 Department of Psychiatry, Lausanne University Hospital and University of Lausanne, Prilly, VD, CH; 22 Department of Psychiatry and Psychotherapy, University Medicine Greifswald, Greifswald MV, DE; 23 Child and Adolescent Psychiatry, Erasmus University Medical Center Rotterdam, Rotterdam, NL; 24 Social and Behavioral Science, Harvard T.H. Chan School of Public Health, Boston, MA, US; 25 Institute of Epidemiology and Social Medicine, University of Münster, Münster, NRW, DE; 26 Department of Genetic Epidemiology in Psychiatry, Central Institute of Mental Health, Medical Faculty Mannheim, Heidelberg University, Mannheim, BW, DE; 27 Department of Medical Epidemiology and Biostatistics, Karolinska Institutet, Stockholm, SE; 28 Psychiatry, Dalhousie University, Halifax, NS, CA; 29 Psychiatry, Kaiser Permanente Northern California, San Francisco, CA, US; 30 Max Planck Institute of Psychiatry, Munich, BY, DE; 31 Neuroscience Therapeutic Area, Janssen Research and Development, LLC, Titusville, NJ, US; 32 Child Health Research Centre, University of Queensland, Brisbane, QLD, AU; 33 Brain and Mind Centre, University of Sydney, Sydney, NSW, AU; 34 Institute for Molecular Bioscience, University of Queensland, Brisbane, QLD, AU; 35 Queensland Brain Institute, University of Queensland, Brisbane, QLD, AU; 36 Department of Psychology, University of Texas at Austin, Austin, TX, US; 37 Population Research Center, University of Texas at Austin, Austin, TX, US; 38 Andres Metspalu, Lili Milani, Tõnu Esko, Reedik Mägi, Mari Nelis & Georgi Hudjashov; 39 Department of Medical & Molecular Genetics, King’s College London, London, UK; 40 Institute for Genomics and Cancer, University of Edinburgh, Edinburgh, UK

## Abstract

Diagnostic criteria for major depressive disorder allow for heterogeneous symptom profiles but genetic analysis of major depressive symptoms has the potential to identify clinical and aetiological subtypes. There are several challenges to integrating symptom data from genetically-informative cohorts, such as sample size differences between clinical and community cohorts and various patterns of missing data. We conducted genome-wide association studies of major depressive symptoms in three clinical cohorts that were enriched for affected participants (Psychiatric Genomics Consortium, Australian Genetics of Depression Study, Generation Scotland) and three community cohorts (Avon Longitudinal Study of Parents and Children, Estonian Biobank, and UK Biobank). We fit a series of confirmatory factor models with factors that accounted for how symptom data was sampled and then compared alternative models with different symptom factors. The best fitting model had a distinct factor for *Appetite/Weight* symptoms and an additional measurement factor that accounted for missing data patterns in the community cohorts (use of Depression and Anhedonia as gating symptoms). The results show the importance of assessing the directionality of symptoms (such as hypersomnia versus insomnia) and of accounting for study and measurement design when meta-analysing genetic association data.

## Introduction

Major depressive disorder (MDD) is a mood disorder characterized by low mood, loss of interest or pleasure (anhedonia), irritable affect, biological symptoms (psychomotor agitation/slowing, altered sleep patterns, changes in appetite or weight), negative thought content, and associated loss of function. To qualify for a diagnosis of major depression, the standard diagnostic classification systems (American Psychiatric Association, 2000, 2013; World Health Organization, 1992) require one of two cardinal symptoms plus at least four other symptoms to be present. Although conceptualized as a single disorder, the diagnostic criterion for MDD can be met with any combination of these other symptoms. For the DSM-5, this entails that there are 227 symptom profiles that would lead to a diagnosis of major depression (Zimmerman et al., 2015). When considering all potential symptom states (such as increasing versus decreasing appetite) the number of possible symptom profiles blooms into the thousands (Fried & Nesse, 2015a).

A single categorical phenotype---that might mask a multitude of separate disorder types---stymies the testing of correlates and treatments. Network analysis has shown that MDD symptoms are not all equally related to each other (Borsboom & Cramer, 2013) and latent class analysis has been used to identify several MDD subtypes with differing patterns of symptoms and differential association with demographic, psychological, and physical health factors (Lamers et al., 2010). However, the potential concealed heterogeneity within the MDD diagnosis does have an upper bound: only around one quarter of the potential symptom profiles are actually observed (Fried & Nesse, 2015a; Zimmerman et al., 2015). This suggests there is both regularity and variation in symptom presentation.

Analysing individual symptoms is one way to unwrap the heterogeneity of MDD (Cai et al., 2020; Fried & Nesse, 2015b). Phenotypic studies have derived and tested factor structures of MDD symptoms (Elhai et al., 2012; Krause et al., 2008, 2010) and twin models have been used to separate genetic from environmental sources of symptom covariance (Kendler et al., 2013). These models grouped symptoms together in two or three factors, which broadly contrast psychological versus somatic symptoms. The primary difference among the proposed two factor structures is whether psychological symptoms including anhedonia and concentration problems group with the cognitive/affective symptoms or with the somatic symptoms. Three factor models have instead posited splitting the psychological symptoms into affective and cognitive components. Clinical subtypes are also part of diagnostic criteria and these have been used to classify depression profiles that are differentially associated with specific clinical, behavioural and biological correlates (Milaneschi et al., 2020; Penninx et al., 2013).

More recently, genetic studies of depressive symptoms have updated the findings from twin models using data from genome-wide association studies (GWAS). A confirmatory factor analysis of genetic covariance estimates obtained from GWAS results on current depressive symptoms showed that a psychological and somatic factor had the best fit to the data (Thorp et al., 2020). The detection of genetic correlates specific to each symptom implies that symptoms may have differing genetic causes and consequences, even if the symptoms themselves are highly genetically correlated.

Understanding the genetic architecture of MDD symptoms is complicated by symptom ascertainment. In clinical samples, symptom data is often only available on affected participants, and is thus conditioned on having been diagnosed with depression. Conditioning data presence on a diagnosis can induce downward bias in correlations amongst the symptoms comprising that diagnosis. However, when symptom data is missing in controls, imputing the absence of the symptom in controls and including them in the analysis has the potential to recapitulate the signal from a case/control analysis rather than reveal genetic variance that is unique to each symptom. In community cohorts, participants are typically screened for the presence of cardinal symptoms (depressed mood and anhedonia) and only participants who report at least one cardinal symptom are assessed for other symptoms of depression, which also leads to high levels of missing symptom data in these cohorts. Moreover, because community samples often contain symptom but not diagnostic information, many GWAS purporting to investigate MDD may actually be better characterized as investigating a broader dysphoria continuum rather than MDD specifically (Flint, 2023). Because community cohorts tend to have a larger sample size than clinical cohorts, meta-analysing all data together therefore has the potential to dilute information on case subtypes.

In this study we sought to uncover the genetic structure of depression symptoms while accounting for how samples were recruited and how symptoms were assessed. We did this by conducting GWAS of individual symptoms of depression, testing factor models to investigate genetic heterogeneity as a function of sample ascertainment (Clinical vs Community) and measurement (with or without screening based on cardinal/gating symptoms). Finally, we assessed the validity of the identified latent factors of depression by estimating genetic correlations with external traits.

Specifically, we conducted GWAS of symptom data in six cohorts and meta-analysed them in groups based on sample ascertainment. The first group (the “Clinical” cohorts) consisted of clinical cases from the Psychiatric Genomics Consortium MDD cohorts, participants from the Australian Genetics of Depression study who were recruited based on depression diagnosis, and participants from Generation Scotland who met DSM criteria for depression. The second group (the “Community” cohorts) consisted of the Avon Longitudinal Study of Parents and Children, Estonian Biobank, and UK Biobank, and thus contained data on participants who were not recruited with respect to depression status. Using the two sets of meta-analysed symptom GWASs, we first constructed and tested factor models that accounted for how the samples were recruited (Clinical versus Community) and how symptoms were assessed (such as gating symptoms in the Community cohorts). After understanding the measurements structure of the symptom GWASs, we then compared alternative factor models for the symptoms based on previous literature and diagnostic specifiers for depressive disorders. Using the best fitting overall model, we tested for shared and specific genetic correlates with other psychiatric, behavioral, and metabolic phenotypes that have known genetic links to MDD.

## Methods

### Samples and symptom measures

We analysed depression symptom data in six studies: the Psychiatric Genomics Consortium, the Australian Genetics of Depression Study, Generation Scotland, the Avon Longitudinal Study of Parents and Children, Estonian Biobank, and UK Biobank. Table 1 describes the number of participants with and without each symptom for each grouping of studies that were analysed. See Supplementary Material for information on genotyping and imputation.

Data from the Psychiatric Genomics Consortium (PGC) was drawn from 23 cohorts in the Wave 1 and Wave 2 datasets of the Major Depressive Disorder Working Group (Major Depressive Disorder Working Group of the Psychiatric GWAS Consortium, 2013; Wray et al., 2018). Symptoms were assessed by trained interviewers using structured diagnostic instruments and DSM checklists. Because information on symptom presence was not available for control participants in most cohorts, participants with a diagnosis of depression were selected for analysis (N = 12,821).

The Australian Genetics of Depression Study (AGDS) (Byrne et al., 2020; Mitchell et al., 2022) is a study of depression and therapeutic response recruited using nationwide prescribing history and through publicity targeting adults who are or had ever been treated for clinical depression (N = 20,689). Symptoms experienced during the participant’s worst period of depression were assessed using the Composite International Diagnostic Interview (CIDI) Short Form (Hickie et al., 2001) and administered through an online questionnaire. Because the study was enriched for participants with a history of being diagnosed with or treated for depression, AGDS was grouped as a Clinical cohort.

Generation Scotland: Scottish Family Health Study (GS:SFHS) is a study of 7,000 families recruited from the general population of Scotland (Smith et al., 2012). Participants who screened reported seeking help for emotional or psychiatric problems were administered an in-person structured interview (Fernandez-Pujals et al., 2015; Smith et al., 2012); and a subset participated in an online follow-up that included a CIDI (Composite International Diagnostic Interview) questionnaire. Symptom data was analysed on participants who met DSM criteria for depression at either time point (N = 3,493).

The Avon Longitudinal Study of Parents and Children (ALSPAC) is a UK-based population birth cohort (Boyd et al., 2013). Participants were from the children sample (N = 13,988) with symptoms present during the last two weeks assessed using the Clinical Interview Schedule Revised (CIS-R) (Lewis et al., 1992) collected during clinical visits at ages 18 and 24. Participants were considered to have had a symptom if they reported it at either measurement occasion.

Estonian Biobank (EstBB) is a population health cohort recruited from medical practitioners in Estonia (Leitsalu et al., 2015). Participants responded to a CIDI questionnaire of depression symptoms during the Mental Health online Survey (MHoS) recontact. Participants were first screened of the presence of low mood or anhedonia and then asked about symptoms during the worst period of depression (N = 84,079).

UK Biobank (UKB) is a population health cohort recruited from general practitioners in the United Kingdom (Sudlow et al., 2015). Lifetime depression symptoms were assessed during online recontact and taken from the CIDI portion of the Mental Health Questionnaire (Davis et al., 2020) (UKB-MHQ, N=157,366) and from assessments of low mood and anhedonia from the baseline touchscreen questionnaire (UKB Touchscreen, N=222,061). For the CIDI, low mood and anhedonia were used as gating symptoms, where participants had to endorse at least one to be asked about the other symptoms.

### Genome-wide association meta-analysis

Genome-wide association study (GWAS) analyses were conducted on each symptom separately in the cohorts (PGC, AGDS, GS:SFHS, ALSPAC, EstBB, UKB-MHQ) on participants who had genetic similarity with each other and the 1000 Genomes European reference. Participants in UKB who clustered with other reference populations were not analysed because sample sizes did not meet the threshold for LD score estimation (N > 5000). See Supplementary Material for more information on the individual study GWASs. We meta-analysed the GWAS summary statistics based on the ascertainment design. PGC, AGDS, and GS:SFHS were meta-analysed together to form the “Clinical” symptom summary statistics; and ALSPAC, EstBB, and UKB-MHQ were meta-analysed together for the “Community” summary statistics. We performed the meta-analyses using Ricopili (Lam et al., 2020) and calculated SNP-based heritability using LD Score Regression (LDSC) (Bulik-Sullivan et al., 2015). For input into LDSC we set the sample size equal to the sum of effective sample sizes of each cohort in the meta-analysis and then specified sample prevalences of 50% (Grotzinger et al., 2022). Symptoms’ population prevalences were estimated for the Clinical cohorts by multiplying the observed sample prevalence by the prevalence of MDD (15%) and for the Community cohorts by multiplying by the proportion of participants in the UKB MHQ sample who were positive on either one the gating symptoms. We assessed significant associations in the meta-analysed summary statistics at *p* < 5 × 10^−8^ / 22 (the number of meta-analyses conducted) or at *p* < 5 χ 10^−8^ with prior association or biological evidence at the locus.

### Confirmatory factor analysis of Genetic Covariance Structure

We fit confirmatory genetic factor analysis models to the meta-analysed ascertainment cohort (i.e., Clinical and Community-based) and UKB Touchscreen summary statistics for each symptom using Genomic SEM (Grotzinger et al., 2019). We first fit a common factor model, where all symptoms load on a single factor as a baseline, using symptoms with a non-negative LDSC heritability (Model A). To explore how sample ascertainment influenced the genetic correlations among the symptoms, we fit a series of models that captured various aspects of the sampling and measurement processes. We then used these results to inform the construction of models that grouped the symptoms based on previous findings and diagnostic criteria. We assessed relative model fit using Akaike Information Criterion (AIC) to pick the best model and absolute model fit with Standardized Root Mean Square Residual (SRMR) to determine how well the model was capturing the genetic correlations among symptoms. We also examined residual correlations to understand what aspects of symptom structure were not being captured. Factor structures are listed in Supplementary Table S4 and illustrated in Supplementary Figure S1.

### Ascertainment/measurement models

The most pertinent measurement difference among the symptoms was which meta-analysed cohorts the symptom came from, so we created a two-factor model where all symptoms from the same cohorts (Clinical or Community) loaded on the same factor (Model B). The next model considered the effect of the cardinal symptoms as gating items in UK Biobank and posited a general MDD factor that all the symptoms loaded on alongside an uncorrelated Gating factor with loadings from just the Community and UKB Touchscreen *low mood* and *anhedonia* symptoms (Model C). The Gating factor would therefore isolate variation associated with differences across the full non-clinical (dysphoria) to clinical spectrum. Symptoms not loading on the gating factor (i.e., those for which data are conditional on the presence of the two gating symptoms) represent variation within the more severe region of the spectrum and are thus more directly comparable to analyses of data from cases only. We then combined the Clinical-Community and Gating models to create a three-factor model (Model D).

### Symptom models

Based off the best measurement model, we then fit models that grouped symptoms into two or three factors based on previous findings from phenotypic, twin, and Genomic SEM models and from diagnostic criteria. The two factor models grouped symptoms into Psychological and Somatic (Model E); Psychological and Neurovegetative (Model F); or Affective and Neurovegetative (Model G) factors (Elhai et al., 2012; Krause et al., 2008, 2010; Thorp et al., 2020). The Affective factor contained symptoms *low mood, feelings of guilt*, and *suicidality*. The Psychological factors broadened the Affective factor to include the symptoms *anhedonia and/or loss of concentration*. The Somatic factor included the *appetite, sleep, fatigue*, and *psychomotor* symptoms. The Neurovegetative factors incorporated the somatic symptoms while also including *loss of concentration* and/or *anhedonia*. A three factor model (Model H) loaded symptoms onto cognitive (*feelings of guilt, loss of concentration, suicidality)*, mood (*low mood, anhedonia, feelings of guilt)*, and neurovegetative (*appetite, sleep, fatigue, psychomotor)* (Kendler et al., 2013).

We also fit factor models that disaggregated symptoms that involved an increasing or decreasing change (*appetite/weight, sleep, psychomotor*). One such model (Model I) was based on previous findings that identified factors for Appetite (*appetite/weight decrease* and *increase)*, vegetative (*hypersomnia, psychomotor slowing, fatigue, concentration)* and Cognitive/Mood (*low mood, anhedonia, insomnia, psychomotor agitation, feelings of guilt, suicidality)* (van Loo et al., 2022). Finally, we considered a three-factor model (Model J) based on diagnostic criteria of melancholic depression (*anhedonia, insomnia, psychomotor agitation, appetite/weight decrease, feelings of guilt*) and atypical depression (*hypersomnia, appetite/weight increase, psychomotor slowing, fatigue*), with the remaining symptoms loading on an Affective/Cognitive factor (*low mood, suicidality, loss of concentration)*.

### Genomic factor meta-analysis

We conducted multivariate meta-analyses of symptoms in Genomic SEM (Grotzinger et al., 2019). Because of low power in some of the Clinical and Community symptoms summary statistics, we were unable to fully test SNP effects on symptom factors. We therefore fit a model with single common factor meta-analysis across well-powered symptoms ($h_{\mathrm{SNP}}^{2}>0,g_{\mathrm{cov}}\;\mathrm{intercept}>1,\bar{\chi^{2}}>1.02$) and tested for SNP heterogeneity at the level of individual symptoms. A genomic factor meta-analysis estimates SNP associations in two structural models: a common pathway model where the SNP is associated with each symptom through its effect on the factor and an independent pathway model where the SNP is associated independently with each symptom, bypassing the common factor. The SNP coefficients from the common pathway model act as a meta-analysis of the symptom summary statistics while accounting for sample overlap. A comparison of fit between the common and independent pathway models yields a heterogeneity statistic for each SNP, Q_SNP_, indicating whether the SNP’s association varies between symptoms.

### Regression Analysis

Using the best fitting models, we tested how the factors were related to correlates of depression. We selected phenotypes that are known to genetically correlate with depression, including psychiatric disorders (anxiety disorder, bipolar disorder, PTSD, schizophrenia); depression defined through clinical ascertainment (major depressive disorder) and through broader or more minimal definitions (major depression); and other health, behavioural, and social phenotypes (see Supplementary Materials for list of studies). We tested whether the other phenotypes had specific genetic correlations with each symptom factor. We did this by first fitting single regressions of a phenotype on each symptom factor. We then compared this to a multiple regression of the phenotype on all symptom factors simultaneously. We used Benjamini–Yekutieli FDR adjustment to correct for multiple testing (Benjamini & Yekutieli, 2001).

## Results

### Genome-wide association and meta-analyses

We conducted GWAS for each symptom separately in all cohorts and meta-analysed within sample ascertainment groups (*Clinical* or enriched cohorts: PGC, AGDS, GS:SFHS; *Community* cohorts: ALSPAC, EstBB, UKB-MHQ) (Supplementary Table S1). Two associations met the stringent multiple testing burden (*p* < 5 × 10^−8^ / 22). One was an intron in *FTO* (ENSG00000140718, alpha-ketoglutarate dependent dioxygenase, a gene involved in food intake) associated with *Weight gain* in the Community cohorts. The other was associated with *Anhedonia* in the Community cohorts and was an intron variant in an uncharacterised non-coding RNA gene (LOC105379109/ENSG00000251574) and in a region previously associated with neuroticism, depression, and subjective well-being.

At the genome-wide significance threshold (*p* < 5 × 10^−8^) there were three associations that were also supported by prior evidence. There were two associations with *Depressed mood* in the Community cohorts: an intron in COMP (ENSG00000105664, cartilage oligomeric matrix protein) also near *CRTC1* (ENSG00000105662, CREB regulated transcription coactivator 1, a gene that regulates metabolism); and an intron in an uncharacterised gene (LOC107986777) regionally associated with depression. An upstream variant for an uncharacterised long intergenic non-protein coding RNA (LINC01938) was associated with Community *Anhedonia* and in a region previously associated with neuroticism and major depressive disorder.

**Figure 1.**
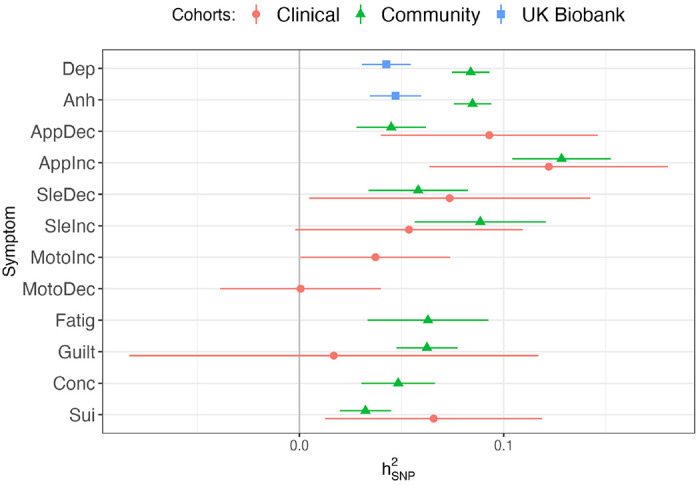
LDSC-estimated heritabilities ($h_{\mathrm{SNP}}^{2}$) on the liability scale of depression symptoms (abbreviations are listed in Table 1) for summary statistics that met inclusion criteria (N_Eff_ > 5000, $h_{\mathrm{SNP}}^{2}>0$). Clinical = PGC + AGDS + GS:SFHS meta-analysis, Community = ALSPAC + EstBB + UKB-MHQ meta-analysis, UK Biobank = UKB-Touchscreen GWAS.

LDSC-estimated heritabilities were primarily in the 0.025–0.1 range (Figure 1, Supplementary Table S3). Many of the symptoms in the Clinical cohorts (*Depressed mood*, *Anhedonia, Fatigue, Concentration)* had negative heritabilities and the psychomotor symptoms from the Community cohorts did not meet the sample size inclusion criteria (N_Eff_ > 5000).

### Confirmatory factor analysis

We brought forward symptoms from the Clinical and Community cohorts meta-analyses and the UKB Touchscreen assessment that had a $h_{\mathrm{SNP}}^{2}$ greater than 0 and sample sizes > 5000 for confirmatory factor analysis (Supplementary Table S4, Supplementary Figure S1a–l).

A common factor model (A) of the symptoms showed poor fit (CI=0.932, SMR=0.169, AIC = 5355). A model (B) with separate factors for Clinical and Community cohort symptoms had slightly poorer fit (AIC = 5369) and yielded a genetic correlation between the two factors of *r_g_* = 0.63±0.14, p = 1.3 ×10^−5^. An alternative model (C) that only split off the Community and UKB-Touchscreen *mood* and *anhedonia* symptoms into an orthogonal factor, capturing these symptoms use as gating items in EstBB and UKB-MHQ, showed substantially improved fit (AIC = 3229). A model (D) combining the sample factors with the orthogonal Gating factor also improved model fit (AIC = 3285) and led to a nominal increase in the genetic correlation between the Clinical and Community factors to *r_g_* = 0.75±0.17, p = 6.9 ×10^−6^.

**Figure 2.**
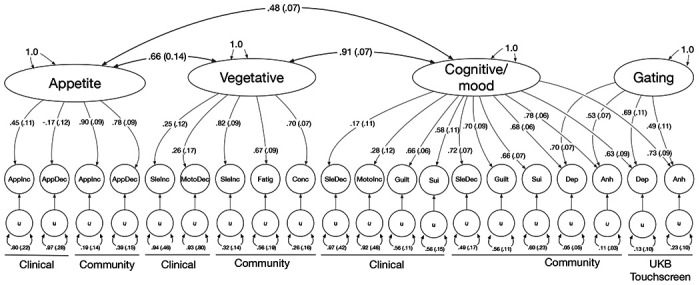
Standardised loadings (standard errors) of factors on symptoms and genetic correlations among factors for the best fitting model (Model I). Symptom abbreviations are listed in Table 1.

We then tested whether models that grouped symptoms together across cohorts fit better than the factor models based on sampling methodology. Because the addition of an orthogonal Gating factor improved model fit so substantially, the symptom-oriented models all included this factor. The best fitting of the symptom models was Model I which included factors capturing Appetite, Vegetative, and Cognitive/Mood symptoms (Figure 2). The Appetite factor had a genetic correlation of *r_g_* = 0.66±0.09 (p = 1.4 ×10^−12^) with the Vegetative factor and *r_g_* = 0.48±0.07 (p = 3.4 × 10^−13^) with the Cognitive/Mood factor, while the Vegetative and Cognitive/Mood factors were more highly correlated, *r_g_* = 0.91±0.07 (p = 7.8 × 10^−40^).

None of the models fully captured the genetic correlations among the symptoms, as indicated by high SRMR (Model D = 0.149, Model I = 0.147). An inspection of the residual genetic correlations (Supplementary Figure S3d) indicated correlations between the same symptoms across the two cohorts (e.g., Clinical *appetite decrease* with Community *appetite decrease*) were not fully represented by the factor structure. We thus tested how adding residual correlations between symptoms that included from both cohorts (*appetite decrease, appetite increase, insomnia, hypersomnia*, and *suicidality)* improved absolute model fit. The addition of these residual correlations lowered SRMR to 0.140.

### Multivariate meta-analysis of symptoms

Because many of the symptom summary statistics were low powered, we were unable to conduct a multivariate meta-analysis using the genetic factors. Alternatively, to test for SNP effects specific to each symptom, we conducted a multivariate meta-analysis of well-powered symptoms summary statistics. The common factor had three genome-wide significant associated variants: an intron variant in BRINP2 (ENSG00000198797, BMP/retinoic acid inducible neural specific 2, a regulator of neuronal differentiation); the upstream variant that was associated with Community *Anhedonia* symptoms; and an intron variant in LRRC37A3 (ENSG00000176809, leucine rich repeat containing 37 member A3) An intron in the FTO gene showed substantial heterogeneity in the common factor meta-analysis (Supplementary Table S7).

### Genetic multiple regression

**Figure 3.**
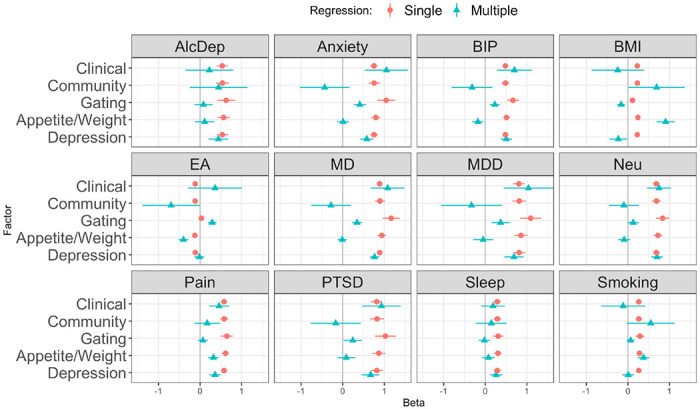
Genetic regression of phenotypes on Clinical and Community cohort factors; and on Gating, Appetite/Weight, and Depression symptom factors. Single genetic regression standardized beta coefficients (green triangles) and multiple genetic regression coefficients, which adjust for the other factors (Clinical-Community adjusted together; Gating-Appetite-Depression adjusted together). AlcDep = alcohol dependence, Anxiety = anxiety disorder, BIP = bipolar disorder, BMI = body-mass index, EA = educational attainment, MD = major depression, MDD = major depressive disorder, Neu = neuroticism, Pain = chronic pain, PTSD = post-traumatic stress disorder, Sleep = long sleep duration, Smoking = cigarettes per day.

To determine whether each MDD symptom factor had specific genetic relationships with twelve phenotypes, we conducted genetic multiple regressions in GenomicSEM using the Clinical–Community sample and Appetite–Vegetative–Cognitive/Mood symptom factor models. Because the Vegetative and Cognitive/Mood symptom factors had a high genetic correlation, we combined these into one factor, which we labelled Depression. We first calculated single regressions of each phenotype on each of the factors separately, where the standardized coefficient indicated the overall shared genetics with each factor. We then then fit a genetic multiple regression with the two models, where the standardized coefficient represents the unique genetic relationship of the phenotype with each factor, after adjusting for shared overlap with the other factors (e.g.., a phenotype’s genetic relationship with the Clinical factor after adjusting for the Community factor, and vice versa; and its genetic relationship with the Appetite factor after adjusting for the Depression and Gating factors, and vice versa). In the single regression (unadjusted) analysis, the genetic relationship of each phenotype with all of the factors were in the same direction with the exception of educational attainment which had a negative relationship with most of the factors (at *p* < 0.0005) but a positive yet non-significant relationship with the Gating factor (Figure 3, Supplementary Table S7). After adjusting for shared overlap with the Clinical factor, the Community factor did not have any specific relationships with the phenotypes, while the Clinical factor had specific, positive genetic correlations with anxiety, bipolar disorder, major depression, major depressive disorder, neuroticism, PTSD, and chronic pain.

When adjusting for the Depression and Gating factors, the factor for the Appetite symptoms had specific genetic correlations with BMI and smoking. The factor for the remaining Depression symptoms was specifically correlated with alcohol dependence, anxiety, neuroticism, and long sleep duration. The Gating factor did not have any specific genetic correlates after adjusting for the other factors, but it did share with the Depression factor positive genetic correlations with bipolar disorder, major depression and major depressive disorder, and PTSD after adjustment for the Appetite factor (Figure 3, Supplementary Table S7).

## Discussion

We used genome-wide association data to analyse the genetic relationships among symptoms of depression based on cohort sampling and symptom content and to estimate whether the genetic factors had specific correlates with other phenotypes. We analysed data from two sets of cohorts: Clinical cohorts that were ascertained to have depression through clinical or interview assessments or were recruited preferentially on a history of treatment for depression; and Community cohorts that were not recruited based on disease status (but for which symptom data was typically conditioned based on endorsement of cardinal gating symptoms). We conducted GWAS of major depression symptoms in each cohort then meta-analysed within the Clinical and Community groups.

We identified loci associated with individual major depression symptoms and with a common genetic factor of the symptoms. Several associations from the individual symptoms and common factor meta-analysis (rs7515828, r rs30266, s6884321, rs150046352) have been identified previously in GWAS of or unipolar depression (EFO ID EFO_0003761) (Sollis et al., 2023) or in meta-analyses of major depressive disorder (Als et al., 2022; Howard et al., 2019; Levey et al., 2021; Wray et al., 2018) SNPs associated with *Appetite / weight increase* have primarily come up in GWAS of body mass index and related traits (Elsworth et al., 2020; Hoffmann et al., 2018; Howe et al., 2022; Yengo et al., 2018) but another SNP in the FTO gene has also been associated with atypical subtypes (Milaneschi et al., 2014).

Many symptoms in the Clinical cohorts had heritability estimates that were zero or negative. This is not unexpected. Selecting individuals based on their phenotype (that is, that they are or have been affected by depression) would result in a sample that has a lower genetic variance for traits that contribute to the determination of the phenotype (Falconer & Mackay, 1996). Additionally, the symptoms with negative heritabilities (*depressed mood, anhedonia, fatigue, concentration problems* in the clinical cohorts) also had high endorsement rates (85–94%), and power to detect heritability is reduced the further the sample prevalence deviates from 50% (Lee et al., 2011). In contrast, the other symptoms had more equal endorsement rates (34–75%) among affected participants. Although the low heritabilities of symptoms from the Clinical cohorts limited the comprehensiveness of alternative factor models that could be tested, we did find congruence between the Clinical and Community cohort symptoms, with a high genetic correlation between their respective factors. We also showed that model fit was substantially improved by modeling the use of cardinal symptoms (*Low mood* and *Anhedonia*) as gating items for surveys of depression symptoms. Among the models that grouped symptoms together without consideration for symptom direction, we found broad support for a split between psychological and somatic symptoms identified in previous phenotypic (Elhai et al., 2012) and genetic (Thorp et al., 2020) analyses. When directional symptoms were portioned out based on diagnostic specifiers, we found that a three-factor model capturing Appetite, Vegetative, and Cognitive/Mood symptoms (van Loo et al., 2022) had the best fit among all models considered. The correlations among the factors indicated that the Vegetative and Cognitive/Mood symptoms should be grouped together, with only the Appetite symptoms making up a possibly different dimension of depression. However, the Clinical cohort symptoms had low loadings in both the sample-based and symptom-based models (except for the Clinical *Appetite/Weight* and *Suicidality* symptoms), and thus the model fit was driven primarily by capturing the structure among the Community cohort symptoms. This observation is consistent with the fact that the Clinical cohorts are more selected than the community cohorts, and that conditioning data presence on a diagnosis can induce downward bias in correlations amongst the symptoms that aggregate to form the diagnosis. Similar attenuation, albeit to a lesser degree, may be expected for items in community samples whose presence was conditioned on endorsement of cardinal symptoms.

Despite these limitations, the Clinical factor was genetically correlated with all the other phenotypes selected for comparison. A multiple genetic correlation analysis showed that the Clinical and Community factors had a shared genetic relationship with alcohol dependence, bipolar disorder, BMI, educational attainment, MDD, chronic pain, sleep, and smoking. The symptom-based factor model showed discriminative validity between the Appetite symptoms and the rest of the symptoms of depression through relationships with correlates of depression. A positive genetic correlation between increase in appetite/weight with BMI has previously been shown with PGC cohorts (Milaneschi et al., 2017) and in UKB (Badini et al., 2022), and our findings show that this result holds even when adjusting for genetic overlap with other symptoms.

Our results demonstrate the challenges and insights associated with considering symptoms of depression separately. In particular, substantial care must be taken to consider how samples are ascertained (clinical versus community recruitment), how symptoms are measured (the use of gating items in symptom inventories), and including assessments of item direction (e.g., insomnia versus hypersomnia) when modelling the genetic structure of depression symptoms. However, the evaluation of direction was limited to a small subset of symptoms and did not include distinctions such, as low versus irritable mood, or included only partial assessments, such as weight but not appetite changes being assessed in UKB. The coverage of features of atypical and melancholic depression was likewise incomplete. For example, several diagnostic features of the atypical specifier were not included, such as mood reactivity, sensitivity to interpersonal rejection, and leaden paralysis. We also only examined subtypes defined by symptom profiles and not other sources of heterogeneity such as onset, life event exposure, or treatment outcomes (Harald & Gordon, 2012) which may also have a differential biological and genetic basis (Beijers et al., 2019; Milaneschi et al., 2020; Nguyen et al., 2022). The strongest genetic associations were between symptoms of weight/appetite change and genes linked to satiety and metabolism. This highlights the need to phenotype somatic symptoms (weight or sleep changes and fatigue) outside of the context of mental health assessments, so that their specific role in depression can be better isolated. Likewise, the use of gating symptoms makes it difficult to fully capture the range of genetic risk between everyday dysphoria and differences among affected individuals. While the results support the idea that depression is heterogeneous, the genetic liability for symptom profiles and comorbidities can be captured in relatively few dimensions.

## Supplementary Material

Supplement 1

2

## Figures and Tables

**Table 1.** Sample size counts and sample prevalences of presence and absence of each symptom for participants used in the genetic analyses. Meta-analysis of Clinical (PGC, AGDS, GenScot), Community (ALSPAC, EstBB, UKB-MHQ) and UKB Touchscreen.

		Cohort *N* Symptom Present : Absent Sample Prevalence		
Symptom	Abbr.	Clinical cohorts meta	Community cohorts meta	UKB Touchscreen
1. Depressed mood	Dep	21681 : 1748 93%	107956 : 99480 52%	71964 : 57130 56%
2. Anhedonia	Anh	24732 : 2801 90%	181113 : 126167 39%	46952 : 80366 37%
3a. Weight loss / decrease in appetite	AppDec	9265 : 14594 39%	39453 : 36497 52%	0 : 0
3b. Weight gain / increase in appetite	AppInc	7902 : 13167 38%	22612 : 36489 38%	0 : 0
4a. Insomnia	SleDec	18917 : 6573 74%	73144 : 19851 79%	0 : 0
4b. Hypersomnia	SleInc	10586 : 11050 49%	20125 : 20055 50%	0 : 0
5a. Psychomotor agitation	MotoInc	10447 : 12372 46%	113 : 3181 3%	0 : 0
5b. Psychomotor slowing	MotoDec	12701 : 11214 53%	299 : 2995 9%	0 : 0
6. Fatigue	Fatig	23941 : 2497 91%	85304 : 16736 84%	0 : 0
7. Feelings of worthlessness / guilt	Guilt	21921 : 3888 85%	61757 : 43570 59%	0 : 0
8. Diminished concentration	Conc	23974 : 2386 91%	75190 : 23416 76%	0 : 0
9. Recurrent thoughts of death or suicide	Sui	18170 : 9609 65%	46984 : 58885 44%	0 : 0

## Data Availability

Primary code is available from the Psychiatric Genomics Consortium (PGC) GitHub Repository (https://github.com/psychiatric-genomics-consortium/mdd-symptom-gwas/) and meta-analysed summary statistics are available for download from the PGC website (https://www.med.unc.edu/pgc/download-results/). Individual-level PGC data is available by application to the PGC Data Access Committee (https://www.med.unc.edu/pgc/shared-methods/). Data from Estonian Biobank (https://genomics.ut.ee/en/content/estonian-biobank), UK Biobank (https://www.ukbiobank.ac.uk) and ALSPAC (http://www.bristol.ac.uk/alspac/) are available to bonafide researchers upon application. Data from AGDS is available for collaboration by contacting NGM (Nick.Martin@qimrberghofer.edu.au).
